# Corrosion of Q235 Carbon Steel in Seawater Containing *Mariprofundus ferrooxydans* and *Thalassospira* sp.

**DOI:** 10.3389/fmicb.2019.00936

**Published:** 2019-05-08

**Authors:** Shiqiang Chen, Hao Deng, Guangzhou Liu, Dun Zhang

**Affiliations:** ^1^Institute of Marine Science and Technology, Shandong University, Qingdao, China; ^2^Key Laboratory of Marine Environmental Corrosion and Bio-fouling, Institute of Oceanology, Chinese Academy of Sciences, Qingdao, China; ^3^Open Studio for Marine Corrosion and Protection, Qingdao National Laboratory for Marine Science and Technology, Qingdao, China

**Keywords:** Q235 carbon steel, EIS, SEM, XPS, microbiologically influenced corrosion

## Abstract

Iron-oxidizing bacteria (IOB) and iron-reducing bacteria (IRB) can easily adhere onto carbon steel surface to form biofilm and affect corrosion processes. However, the mechanism of mixed consortium induced carbon steel corrosion is relatively underexplored. In this paper, the adsorptions of IOB (*Mariprofundus ferrooxydans, M. f.*), IRB (*Thalassospira* sp., *T*. sp.) and mixed consortium (*M. f.* and *T*. sp.) on surface of Q235 carbon steel and their effects on corrosion in seawater were investigated through surface analysis techniques and electrochemical methods. Results showed that local adhesion is a typical characteristic for biofilm on surface of Q235 carbon steel in *M. f*. and mixed consortium media, which induces localized corrosion of Q235 carbon steel. Corrosion rates of Q235 carbon steel in different culture media decrease in the order: *r*_*M.f.*_ > *r*_mixed consortium_ > *r*_*T*.__sp._ > *r*_sterile_. The evolution of corrosion rate along with time decreases in *M. f.* medium, and increases then keeps table in both *T*. sp. and mixed consortium media. Corrosion mechanism of Q235 carbon steel in mixed consortium medium is discussed through analysis of surface morphology and composition, environmental parameter, and electrochemical behavior.

## Introduction

Carbon steel, as a common material in marine engineering, is vulnerable to corrosion. Microorganisms is one of the significant factors affected the metal corrosion process in marine environment, i.e., more than 20% of corrosion related failures are attributed directly or indirectly to microbiologically influenced corrosion (MIC) (Bhandari et al., 2015). Among the corrosive microbes, iron reducing bacteria (IRB) and oxidizing bacteria (IOB) are two kinds of special microorganisms using iron as an electron acceptor and donor, respectively, (Byrne et al., 2015). IRB combine reduction of Fe(III) with oxidation of organic matter or H_2_ for energy conservation, i.e., IRB readily use dissolved Fe(III) complexes or short-range-ordered minerals (e.g., ferrihydrite) and even magnetite as terminal electron acceptors (Pan et al., 2017; Fortney et al., 2018). IOB grow with Fe(II) or H_2_ as the electron donor coupled to the reduction of oxygen in environments at acidic and circumneutral pH values (Weber et al., 2006; McBeth and Emerson, 2016). In marine environment, Fe^2+^ and Fe^3+^ compounds, i.e., Fe_3_O_4_, FeOOH, Fe_2_O_3_, FeS, etc., are widely distributed, especially for around the marine engineering material based on steel (Qian et al., 2018; Chen and Zhang, 2019), which provides favorable conditions for the survival of these two bacteria. Meanwhile, they can easily adhere onto steel surface to form biofilm and affect corrosion processes (Wang et al., 2012; Li et al., 2016).

Mechanisms of IOB or IRB induced corrosion have been extensively studied. IOB could produce dense deposits made up of intact and/or the partly degraded remains of bacterial cells mixed with amorphous hydrous ferric oxides/hydroxides, which resulted in a crevice effect and induced pitting corrosion of stainless steel (Xu et al., 2007; Starosvetsky et al., 2008; Lee et al., 2013). Additionally, corrosion of carbon steel was promoted due to that Fe^3+^ produced from IOB metabolism can rapidly oxidize Fe^0^ to Fe^2+^ (Wang et al., 2014; Liu et al., 2016; Liu H. et al., 2018). The relationship of IRB to corrosion was not straightforward, i.e., IRB may enhance corrosion under some circumstances, or have a passivating effect on corrosion in others (Duan et al., 2008; Mehanna et al., 2008; Esnault et al., 2011; Schutz et al., 2014, 2015; Cote et al., 2015; Starosvetsky et al., 2016). Corrosion acceleration induced by IRB was mainly related to that the reduction of insoluble ferric compounds to soluble ferrous ion facilitated the removal of protective corrosion products on steel surface (Esnault et al., 2011; Starosvetsky et al., 2016), and bio-oxidation of H_2_ resulted in high anodic dissolution rate of steel (Schutz et al., 2014, 2015). IRB induced corrosion-inhibition was always linked to a modification of the environmental conditions at the metal/solution interface by biological activity, i.e., formation of green rust or an iron (II) phosphate layer in biofilm contained IRB could prevent the corrosive agents from reaching steel surface (Duan et al., 2008; Cote et al., 2015). Based on above results, the mechanisms of IOB or IRB have been studied in-depth, but the joint effects of IOB and IRB on metal corrosion are poorly known. It has been widely accepted that the redox-induced cycling of iron is primarily controlled by combined effects of IOB and IRB in environment (Weber et al., 2006; Lee et al., 2013; Byrne et al., 2015). Investigators have found that the presence of mixed consortium (IOB and IRB) caused a measurable loss from the surface of carbon steel (Lee et al., 2013). However, the mechanism of mixed consortium induced carbon steel corrosion, i.e., surface morphology, corrosion products composition, environmental parameters and electrochemical behaviors, is relatively underexplored.

In this paper, the adsorptions of IOB (*M. f.*), IRB (*T*. sp.) and mixed consortium (*M. f*. and *T*. sp.) on surface of Q235 carbon steel in seawater were investigated by scanning electron microscope (SEM) and energy dispersive spectrum (EDS). Environmental parameters and weight loss in different culture media were measured. Open circuit penitential (OCP) and electrochemical impedance spectroscopy (EIS) were applied to investigate electrochemical information. X-ray photoelectron spectroscopy (XPS) were used to study surface composition of Q235 carbon steel. Finally, the corrosion mechanism of mixed consortium induced corrosion of Q235 carbon steel in seawater was discussed.

## Materials and Methods

### Materials

In this paper, Q235 carbon steel coupons with the shape of disk (10 mm diameter) were used for electrochemical measurements and surface analysis, and cut from Q235 carbon steel plate (wt. %, 0.1 C, 0.4 Mn, 0.12 Si, 0.02 S, 0.05 P, and Fe balance). The Q235 carbon steel electrode were manufactured through embedding in a mold of non-conducting epoxy resin with 0.785 cm^2^ left exposed. Prior to each experiment, the exposed surfaces of all coupons were sequentially polished with a series of mesh silicon carbide emery papers (400, 800, 1200, and 2000) to smoothen. They were then rinsed with deionized water, degreased with absolute ethyl alcohol, and dried with pure nitrogen (>99.99%). Finally, all the coupons were sterilized by exposing to ultraviolet radiation for 30 min.

### Bacterial Culture

Iron-oxidizing bacteria, i.e., *M. f*. (ATCC BAA-1020), were purchased from American Type Culture Collection (ATCC). IRB, i.e., *T*. sp., were isolated from marine sludge collected from South China Sea through a selective culture medium, which is contained (g/L, natural seawater): 0.5 g KH_2_PO_4_, 0.5 g Na_3_NO_3_, 0.2 g CaCl_2_, 0.5 g MgSO_4_, 0.5 g (NH_4_)_2_SO_4_, 24.0 g ammonium ferric citrate. In order to create a same system for further tests, *M*. *f*., *T*. sp., and mixed consortium were cultivated using a same medium. This was prepared by the following procedures: a medium contained (per 1 L seawater) 1 g NH_4_Cl, 0.5 g K_2_HPO_4_, 3 g Na_3_C_6_H_6_O_7_ ⋅ 2H_2_O, and 0.84 g NaHCO_3_ was autoclaved at 121°C for 20 min. After cooling down in air to ambient temperature, the pH value of culture medium was adjusted to 7.0 ± 0.1 using 1 M NaOH or HCl solution. 1 mL Wolf vitamin and trace elements was added in culture medium through 0.2 μm millipore filter, and then the sterilized Q235 carbon steel coupons were added to culture medium under aseptic condition. In this work, because *M*. *f*. and *T*. sp. were microaerophilic and facultative anaerobic microbes, respectively, the DO concentration was adjusted to 2.4 ± 0.1 mg/L through pumping into pure nitrogen (>99.99%) to ensure the survival of these two bacteria. Finally, the culture medium inoculated with *M*. *f*., *T*. sp., or mixed consortium was cultivated in an electric thermostat box (MJX-280, Ningbo Jiangnan Instrument Factory) at 30°C. In culture medium, Na_3_C_6_H_6_O_7_ ⋅ 2H_2_O and corrosion products of Q235 carbon steel served as carbon and iron sources, respectively, for the bacterial growth.

### Environmental Parameter and Weight Loss Measurement

The environment parameter, weight loss and electrochemical tests were performed in a 500 ml wild-mouth bottle with 400 ml media. Concentration of dissolved oxygen (DO) and pH in *M*. *f*., *T*. sp. or mixed consortium media for different times were detected everyday by a dissolved oxygen meter (Thermo Orion 5-Star; Thermo Fisher Scientific Inc., Massachusetts, United States) and a pH meter (PHS-3C; INESA Scientific Instruments Co., Ltd., Shanghai, China), respectively. The weight loss of each coupon was obtained by a XSE analytical balance (METTLER TOLEDO, 0.01 mg/0.1 mg). At different duration time, three coupons were taken out and cleaned according to the standard of ASTM G1-2003.

### Electrochemical Experiments

Open circuit penitential and EIS were measured by using CHI 604D (CH Instruments, Inc.) electrochemical workstation in a three-electrode system. Q235 carbon steel, graphite sheet (surface area is about 4.91 cm^2^), and Ag/AgCl electrode are working, counter and reference electrodes, respectively. Each impedance spectrum was measured at OCP under excitation of a sinusoidal wave with an amplitude of 5 mV, and within a frequency range of 1 × 10^5^ to 1 × 10^-2^ Hz. The results of EIS were analyzed by Zsimpwin software. All electrochemical experiments were conducted at 25 ± 2°C.

### Surface and Component Analysis

The micromorphology of Q235 carbon steel surface was obtained by using a SEM (Hitachi S-3400N), and the elemental distribution of carbon was obtained through the coupled EDS. Before observation, coupons were first fixed with 2.5% glutaraldehyde in a phosphate buffer solution (pH 7.3–7.4) for 2 h, washed with phosphate buffer solution for three times, rinsed with deionized water for another three times, and then dehydrated with using an ethanol gradient (30, 50, 70, 90, and 100 vol. % for 15 min each). Finally, they were supercritically dried, and coated with gold.

Chemical composition information of Q235 carbon steel in *M*. *f*., *T*. sp., and mixed consortium media for 11 days was obtained by XPS (Thermo Fisher Scientific ESCALAB 250, Al Kα radiation). The high-resolution spectra were analyzed through a deconvolution fitting procedure using the XPS Peak-Fit 4.1 software.

## Results and Discussion

### Effects of Bacteria on Environmental Parameters

**FIGURE 1:**
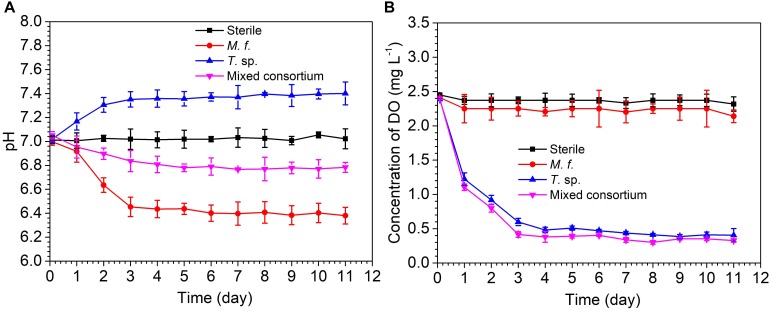
The evolution of **(A)** pH and **(B)** concentration of dissolve oxygen in sterile, *M*. *f*., *T*. sp., and mixed consortium media along with time.

Figure 1 shows the evolution of pH and DO concentration in sterile, *M*. *f*., *T*. sp., and mixed consortium media along with time. As shown in Figure 1A, pH in sterile medium keeps stable at seven for different times. The evolution of pH in *M*. *f*., *T*. sp., and mixed consortium media shows different trends along with time. In *M*. *f*. medium, pH decreases and then keeps stable at 6.4 along with time, which may be due to that Fe^3+^ produced from the metabolism of *M*. *f*. reacts with H_2_O to form insoluble Fe(OH)_3_ and H^+^ (Weber et al., 2006). However, in *T*. sp. medium, pH increases and then keeps stable at 7.4 along with time, which may be due to that Fe^3+^ oxides are reduced by *T*. sp., and OH^-^ as by-products is formed. In mixed consortium medium, pH decreases slightly, and then keeps stable at 6.8, indicating that pH is mainly affected by the presence of *M*. *f*.

The evolution of DO concentration in different culture media also shows different trends along with time. In sterile and *M*. *f*. media, concentration of DO slightly decreases and then keeps stable at 2.37 and 2.25 mg/L, respectively (Figure 1B). This is due to that corrosion of carbon steel consumes little oxygen in sterile medium, and the metabolism of microaerophilic *M*. *f*. results in lower concentration of DO than that in sterile medium. However, in *T*. sp. and mixed consortium media, DO concentration remarkably decreases and then keeps stable at 0.4 mg/L along with time, which may be due to that the facultative *T*. sp. consume the oxygen in culture medium. These behaviors indicate that DO concentration in mixed consortium medium is mainly affected by the presence of *T*. sp.

### Analysis of OCP and Weight Loss of Q235 Carbon Steel

**FIGURE 2:**
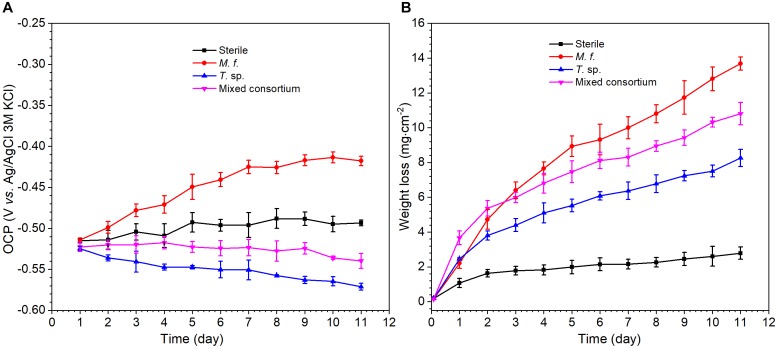
The evolution of **(A)** OCP and **(B)** weight loss of Q235 carbon steel in sterile, *M*. *f*., *T*. sp., and mixed consortium media along with time.

Figure 2 shows the evolution of OCP and weight loss of Q235 carbon steel in different media along with time. As shown in Figure 2A, there are different evolution trends for the OCP in sterile, *M*. *f*., *T*. sp., and mixed consortium media. In sterile and *M*. *f.* media, OCP increases to -0.49 and -0.42 V, respectively, after 11 days of exposure. However, in *T*. sp. and mixed consortium media, OCP decreases with time, and to -0.57 and -0.54 V, respectively, after 11 days of exposure. These behaviors indicate that the evolution of OCP of Q235 carbon steel in mixed consortium medium is closely related with the presence of *T*. sp. In *M*. *f*., *T*. sp., and mixed consortium media, the average weight loss is larger than that in sterile medium after 1 day of exposure, indicating that the presence of bacteria promotes the corrosion of Q235 carbon steel (Figure 2B). Additionally, the average weight loss (WL) in different culture media increases in the order: *WL*_sterile_ < *WL*_*T*_. _sp._ < *WL*_mixedconsortium_ < WL_*M.f.*_ after 2 days of exposure, especially on the 11th day of exposure, the average weight loss in sterile, *T*. sp., mixed consortium, and *M*. *f*. media is 2.78, 8.26, 10.81, and 13.69 mg cm^-2^, respectively (Figure 2B). This indicates that corrosion rates of Q235 carbon steel in different culture media decrease in the order: *r*_*M.f.*_ > *r*_mixedconsortium_ > *r*_*T*.__sp._ > *r*_sterile_.

### Analysis of EIS

**FIGURE 3:**
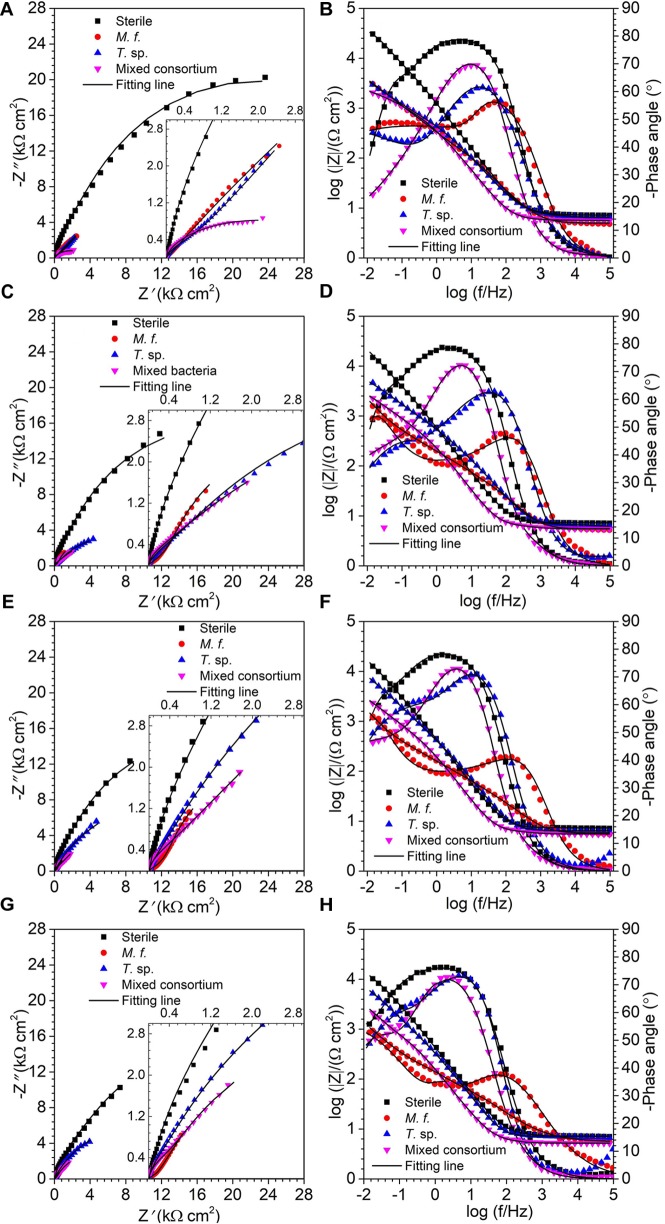
EIS, **(A,C,E,G)** Nyquist and **(B,D,F,H)** Bode plots, of Q235 carbon steel in sterile, *M*. *f*., *T*. sp., and mixed consortium media for **(A,B)** 1, **(C,D)** 4, **(E,F)** 7, and **(G,H)** 11 days.

Figure 3 shows the EIS spectra of Q235 carbon steel in sterile, *M*. *f*., *T*. sp., and mixed consortium media along with time. As shown in Figure 3, a big capacitance arc and two wave peaks are observed in Nyquist and Bode plots, repectively, for Q235 carbon steel in different culture media for different time (Figure 3). These behaviors indicate the presence of two processes, i.e., electron transfer process and film adsorption, which control corrosion process. The diameters of capacitive arcs in bacterial medium are smaller than that in sterile medium. These behaviors indicate that corrosion of Q235 carbon steel is promoted due to the presence of bacteria.

**FIGURE 4:**
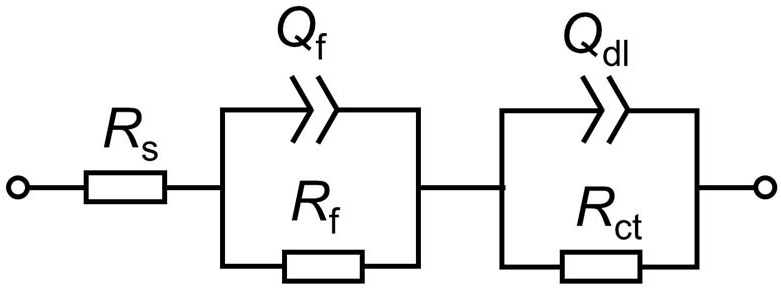
The equivalent circuit model used to fit the EIS data in Figure 3.

Based on above analysis, the EIS in different media is fitted by using an equivalent circuit contained two time-constants, as shown in Figure 4. In the equivalent circuit, *Q* is constant phase element (CPE) and given by:

(1) $$Z_{CPE}=\frac{1}{Y_{0}{\left(j\omega \right)}^{n}}$$

where *Y*_0_ is a parameter related to capacitance, *ω* is angular frequency, *j* is imaginary number, and *n* is exponential term related to roughness of electrode surface. *R*_s_, *R*_f_, and *R*_ct_ indicate resistances of electrolyte, surface film and charge transfer, respectively. *Q*_f_ and *Q*_dl_ are CPEs of surface film and electrical double layer, respectively. The fitting results in different media are listed in Table 1. As shown in Table 1, except for the 1st day of exposure, the value of *R*_ct_ in different culture media increases in the order: *R*_ct_(*M*. *f*.) < *R*_ct_(mixed consortium) < *R*_ct_(*T*. sp.) < *R*_ct_(sterile). These behaviors mean that corrosion of carbon steel in bacterial media, i.e., *M*. *f*., *T*. sp. and mixed consortium media, is promoted, and corrosion rate is biggest in *M*. *f*. medium after 1 day of exposure. Along with immersion time, the value of *R*_ct_ in sterile, *T*. sp., and mixed consortium media increases with time, while in *M*. *f*. medium decreases with time. These behaviors indicate that corrosion is inhibited in sterile medium along with the immersion time, which may be due to the accumulation of corrosion products on carbon steel surface. This agrees with the results of weight loss (Figure 2B). The corrosion rate decreases along with time (Table 1), and the OCP shifts to negative direction with time in both *T*. sp. and mixed consortium media (Figure 2A). This indicates that cathodic reaction process is inhibited, which may be due to the decrease of DO concentration in *T*. sp. and mixed consortium media (Figure 1B). Corrosion is promoted along with immersion time, and the OCP shifts to positive direction with time in *M*. *f*. medium (Figure 2). This means that cathodic reaction process is promoted, which may be due to the decrease of pH, and the high concentration of DO in *M*. *f*. medium (Figure 1). From the above results, the evolution of corrosion rate of Q235 carbon steel in mixed consortium medium is mainly influenced by the presence of *T*. sp.

**Table 1:** The fitting parameter of EIS data of Q235 carbon steel in sterile, *M*. *f*., *T*. sp., and mixed consortium media for different times.

Time/medium		*R*_s_ (Ω cm^2^)	*Q*_f_		*R*_f_ (kΩ cm^2^)	*Q*_dl_		*R*_ct_ (kΩ cm^2^)
					
			*Y*_0_ (10^-4^ Ω^-1^ ⋅ cm^-2^ ⋅ s*^n^*^1^)	*n_1_*		*Y*_0_ (10^-4^ Ω^-1^ ⋅ cm^-2^ ⋅ s*^n^*^2^)	*n*_2_	
1 day	Sterile	9.37 ± 0.07	1.21 ± 0.02	0.90 ± 0.003	22.60 ± 2.94	1.03 ± 0.22	0.98 ± 0.08	36.54 ± 4.46
	*M*. *f*.	5.81 ± 0.09	2.30 ± 0.52	0.82 ± 0.006	0.08 ± 0.01	9.04 ± 0.16	0.63 ± 0.01	13.63 ± 1.50
	*T*. sp.	8.23 ± 0.10	3.77 ± 0.16	0.75 ± 0.004	1.32 ± 0.13	27.08 ± 1.07	0.75 ± 0.05	10.21 ± 0.05
	Mixed consortium	7.23 ± 0.02	3.29 ± 0.07	0.90 ± 0.004	0.59 ± 0.08	8.21 ± 0.50	0.47 ± 0.03	4.55 ± 0.45
4 days	Sterile	9.29 ± 0.03	2.54 ± 0.02	0.91 ± 0.002	12.43 ± 1.26	1.43 ± 0.17	0.80 ± 0.05	39.62 ± 3.78
	*M*. *f*.	6.10 ± 0.17	7.94 ± 0.07	0.61 ± 0.01	0.61 ± 0.05	23.49 ± 1.72	0.65 ± 0.01	5.10 ± 0.73
	*T*. sp.	8.18 ± 0.06	1.47 ± 0.08	0.85 ± 0.07	0.41 ± 0.05	4.91 ± 0.08	0.53 ± 0.01	14.41 ± 3.13
	Mixed consortium	7.19 ± 0.04	5.74 ± 0.14	0.90 ± 0.005	0.83 ± 0.09	12.78 ± 0.95	0.60 ± 0.04	8.25 ± 1.84
7 days	Sterile	9.37 ± 0.03	3.33 ± 0.03	0.91 ± 0.002	8.65 ± 1.53	1.32 ± 0.18	0.66 ± 0.06	54.68 ± 5.44
	*M*. *f*.	6.25 ± 0.17	12.26 ± 0.69	0.55 ± 0.01	0.59 ± 0.05	27.38 ± 2.29	0.62 ± 0.01	4.55 ± 0.49
	*T*. sp.	8.01 ± 0.08	6.81 ± 0.40	0.91 ± 0.03	0.44 ± 0.01	6.04 ± 0.37	0.84 ± 0.05	15.20 ± 1.52
	Mixed consortium	7.14 ± 0.05	11.73 ± 1.98	0.92 ± 0.03	0.56 ± 0.09	22.43 ± 0.57	0.84 ± 0.03	8.87 ± 0.08
11 days	Sterile	9.29 ± 0.05	1.31 ± 0.11	0.92 ± 0.003	7.06 ± 0.68	3.02 ± 0.11	0.76 ± 0.01	56.20 ± 2.16
	*M*. *f*.	6.36 ± 0.14	18.38 ± 0.82	0.50 ± 0.007	0.71 ± 0.07	26.72 ± 2.81	0.60 ± 0.01	3.32 ± 0.57
	*T*. sp.	8.90 ± 0.09	9.28 ± 0.92	0.93 ± 0.04	0.50 ± 0.02	9.02 ± 0.95	0.87 ± 0.06	16.34 ± 1.38
	Mixed consortium	6.65 ± 0.03	18.31 ± 1.49	0.92 ± 0.02	0.49 ± 0.05	25.41 ± 0.80	0.83 ± 0.02	9.02 ± 0.06


### Analysis of Micromorphology

**FIGURE 5:**
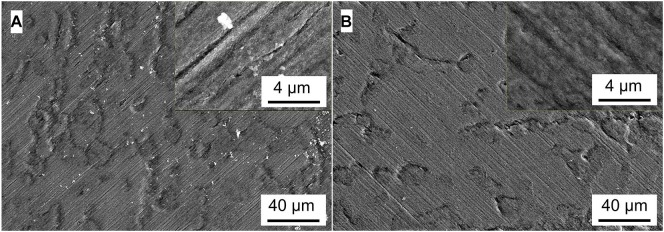
SEM images of Q235 carbon steel **(A)** before and **(B)** after removing corrosion products film in sterile medium for 11 days.

**FIGURE 6:**
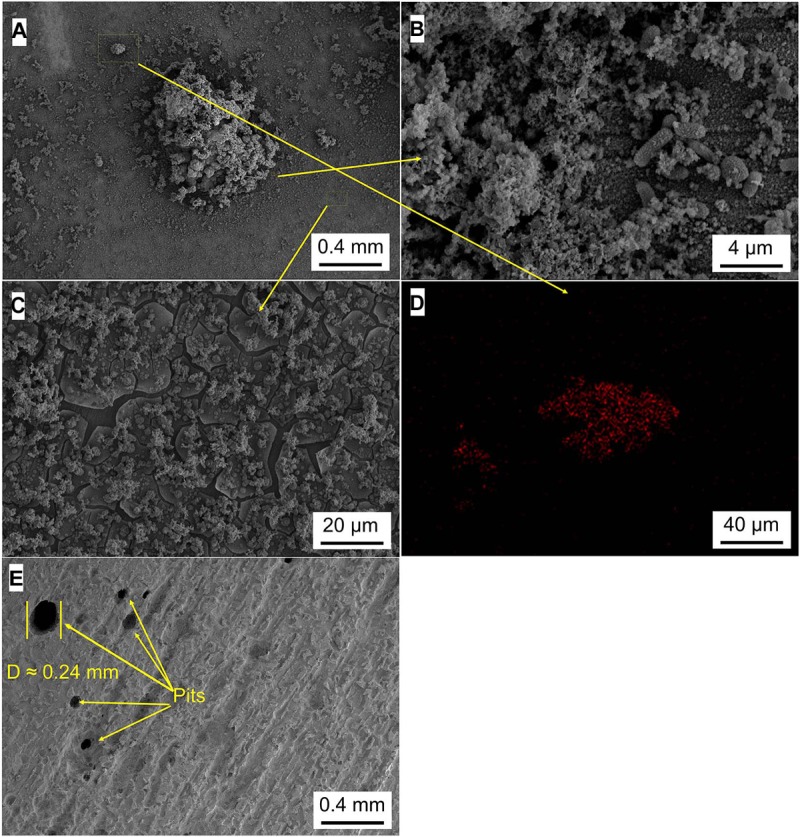
SEM images of Q235 carbon steel **(A–C)** before and **(E)** after removing corrosion products film in *M*. *f*. medium for 11 days, and **(D)** elemental carbon distribution map.

**FIGURE 7:**
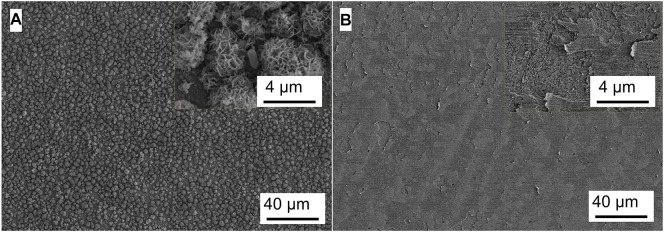
SEM images of Q235 carbon steel **(A)** before and **(B)** after removing corrosion products film in *T*. sp. medium for 11 days.

**FIGURE 8:**
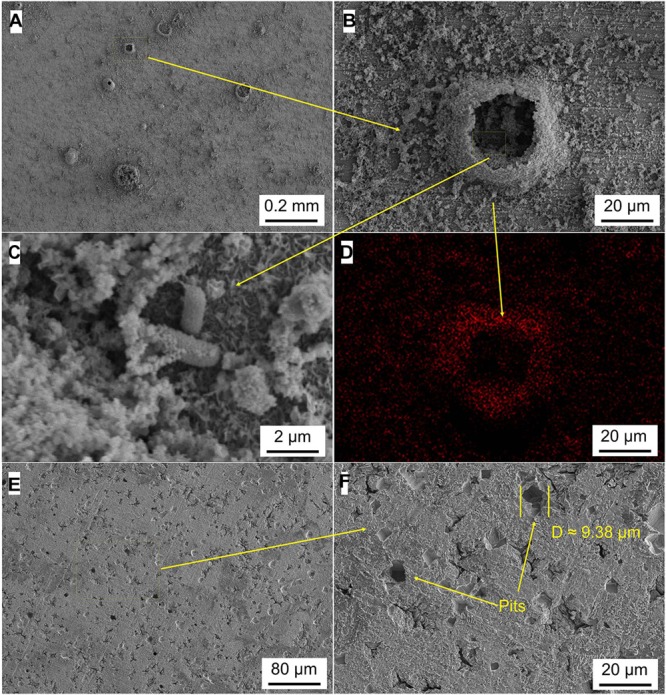
SEM images of Q235 carbon steel **(A–C)** before and **(E,F)** after removing corrosion products film in mixed consortium medium for 11 days, and **(D)** elemental carbon distribution map.

Figure 5–8 show the surface micromorphology of Q235 carbon steel in different media for 11 days. As shown in Figure 5, a compact film adheres on surface of Q235 carbon steel after 11 days of exposure, and after removing corrosion products, uniform corrosion is the main corrosion form. Based on the results in Figure 3, this compact film protects the Q235 carbon steel in sterile medium. In *M*. *f*. medium, the morphology of Q235 carbon steel is obviously different with that in sterile medium, i.e., a huge local cluster coupled with some smaller ones is observed (Figure 6A), some bacterial cells adhere around the cluster (Figure 6B), and EDS distribution map shows the presence of plenty of elemental carbon in the cluster (Figure 6D). These behaviors indicate that biofilm is the main component of these cluster, and heterogeneous distribution is the typical form for *M*. *f*. biofilm on Q235 carbon steel surface (Figure 6A,C,D). After removing the corrosion products and biofilm, several pits are observed (Figure 6E), indicating pitting corrosion is the main form for Q235 carbon steel in *M*. *f*. medium. In *T*. sp. medium, surface morphology is uniform, and lot of granular corrosion products adhere onto the surface of Q235 carbon steel (Figure 7A). After removing the corrosion products, there are many very small pits on surface of Q235 carbon steel (Figure 7B), indicating that uniform corrosion is the main form for Q235 carbon steel in *T*. sp. medium. In mixed consortium medium, the surface morphology is similar with that in *M*. *f*. medium, i.e., there are some local clusters on surface of Q235 carbon steel (Figure 8A), bacterial cells are observed in cluster (Figure 8C), and EDS distribution presents plenty of elemental carbon in these cluster (Figure 8D). These behaviors indicate that the main component of cluster is biofilm, and heterogeneous distribution is the typical form for the biofilm on Q235 carbon steel in mixed consortium medium (Figure 8A,B,D). After removing the corrosion products and biofilm, there are a lot of pits on surface of Q235 carbon steel (Figure 8E,F), meaning that localized corrosion is the main form for Q235 carbon steel in mixed consortium medium. These characteristics, i.e., morphology of biofilm and corrosion form, are similar with that in *M*. *f*. medium, but the degree of localized corrosion in mixed consortium medium is slighter than that in *M*. *f*. medium, i.e., the diameters of typical pits are 240 and 9.38 μm in *M*. *f*. and mixed consortium media, respectively, and based on the statistic, the average diameters of pits are 105 and 8.79 μm in *M. f.* and mixed consortium media, respectively (Figure 6E, 8F). These behaviors indicate that biofilm morphology and corrosion form in mixed consortium medium are mainly affected by the presence of *M*. *f*., and the introduction of *T*. sp. inhibits the corrosion rate of localized corrosion of Q235 carbon steel.

### Analysis of XPS

**FIGURE 9:**
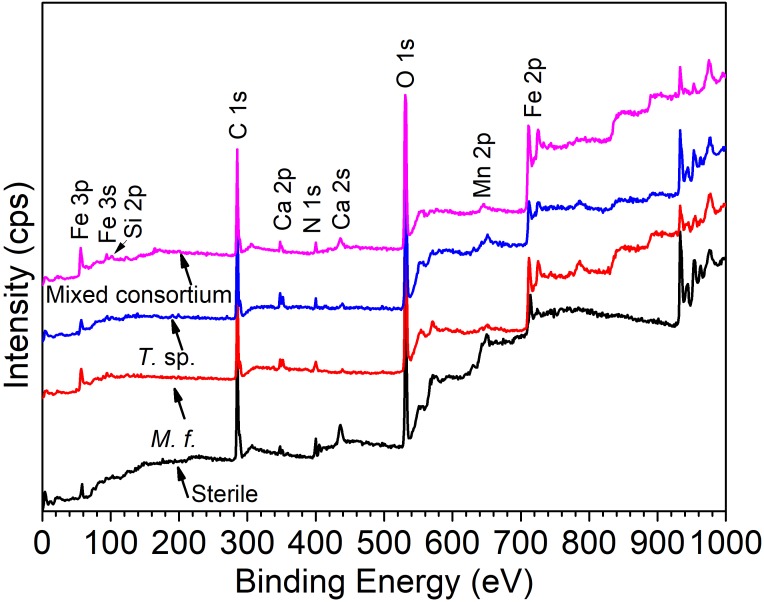
The XPS spectra of Q235 carbon steel in sterile, *M*. *f*., *T*. sp., and mixed consortium media for 11 days.

Figure 9 shows the survey spectra of Q235 carbon steel in sterile, *M*. *f*., *T*. sp., and mixed consortium media for 11 days. As shown in Figure 9, in all media, the following elements dominate on surface of Q235 carbon steel: Fe, C, O, N, Si, Ca, and Mn. In sterile medium, elemental C and N are mainly derived from the adsorption of organic substances from culture medium, and in bacterial media, i.e., *M*. *f*., *T*. sp., and mixed consortium media, mainly belonged to the biofilm. Elemental Si and Ca are from the culture medium, and elemental Mn is from the alloying element of Q235 carbon steel.

**FIGURE 10:**
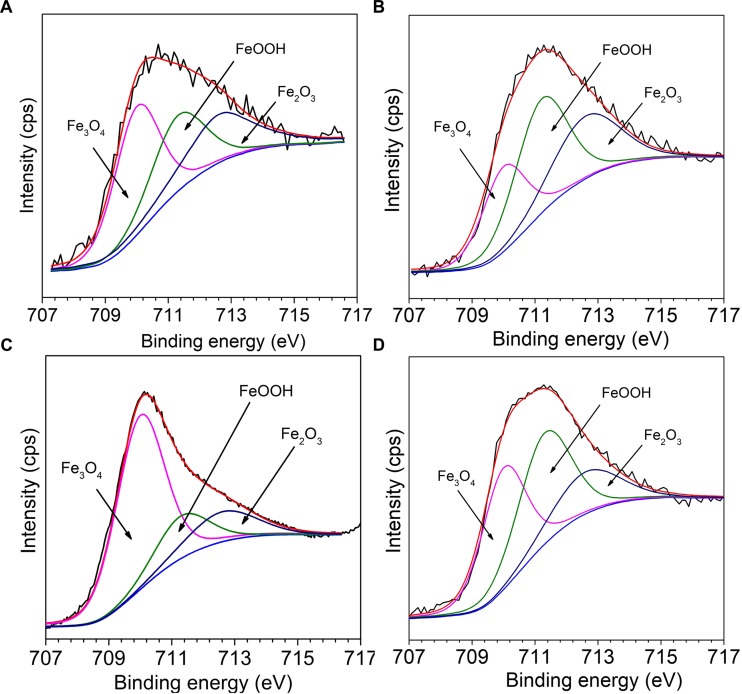
The Fe 2p_3/2_ high resolution spectra of Q235 carbon steel in **(A)** sterile, **(B)** *M*. *f*., **(C)** *T*. sp., and **(D)** mixed consortium media for 11 days.

Figure 10 presents the fitting results of Fe 2p_3/2_ of Q235 carbon steel in sterile, *M*. *f*., *T*. sp., and mixed consortium media for 11 days. The deconvolutions of all Fe 2p_3/2_ spectra of Q235 carbon steel in sterile, *M*. *f*., *T*. sp., and mixed consortium media are fitted into three peaks (Figure 10A–D), i.e., the positions of the peaks are at 710.15 eV, 711.5 eV, and 712.8 eV, which are attributed to Fe_3_O_4_, FeOOH, and Fe_2_O_3_, respectively (Qi et al., 2016; Refait et al., 2016; Blackwood et al., 2017). Obviously, Fe oxides are the main components of corrosion products for Q235 carbon steel, which are same with each other in different media. Thus, it can be inferred that a series of subsequent reactions can occur in mixed consortium medium and generate FeOOH, Fe_2_O_3_, and Fe_2_O_3_:

(2) $$Fe\to Fe^{2+}+2e$$

(3) $$Fe^{2+}\;\overset{IOB}{\underset{IRB}{⇄}}\;Fe^{3+}$$

(4) $$Fe^{3+}+2H_{2}O\to FOOH+3H^{+}$$

(5) $$2FOOH+Fe^{2+}\to Fe_{3}O_{4}+2H^{+}$$

(6) $$Fe_{3}O_{4}\overset{IOB}{\underset{IRB}{⇄}}\;Fe_{2}O_{3}$$

The relative content (RC) of corrosion products components in different media is further analyzed, and the results are listed in Table 2. As shown in Table 2, the RCs of FeOOH and Fe_2_O_3_ in different media decreases in the order: RC_*M.f.*_ (73%) > RC_mixedconsortium_ (62%) > RC_sterile_ (58%) > RC_*T*.__sp._ (34%), and the order of RC of Fe_3_O_4_ is reversed with that of FeOOH and Fe_2_O_3_, i.e., RC_*T*.__sp._ (66%) > RC_sterile_ (42%) > RC_mixedconsortium_ (38%) > RC_*M.f.*_ (27%). This indicates that the RCs of Fe^3+^ and Fe^2+^ in corrosion products are the biggest in *M*. *f*. and *T*. sp. media, respectively. This is derived from the metabolism of bacteria, i.e., *M*. *f*. and *T*. sp. conduct iron oxidization and reduction, respectively. In consortium medium, the RCs of Fe^3+^ and Fe^2+^ are between that in *M*. *f*. and *T*. sp. media, which indicates that RCs of components of corrosion products of Q235 carbon steel in mixed consortium are influenced by the presence of these two bacteria.

**Table 2:** The RC of components of corrosion products on surface of Q235 carbon steel in sterile, *M*. *f*., *T*. sp., and mixed consortium media for 11 days.

Components of corrosion products	Position (eV)	Sterile (At. %)	*M. f*. (At. %)	*T*. sp. (At. %)	Mixed consortium (At. %)
Fe_3_O_4_	710.15	42	27	66	38
FeOOH	711.5	29	43	18	41
Fe_2_O_3_	712.8	29	30	16	21


### Corrosion Mechanism Model

**FIGURE 11:**
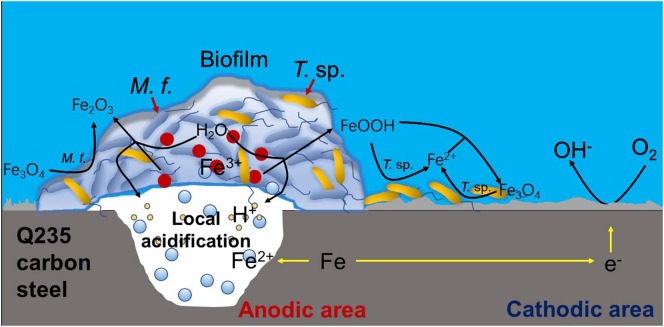
The corrosion mechanism model of Q235 carbon steel in mixed consortium medium.

Based on above analysis, a corrosion mechanism model of Q235 carbon steel in mixed consortium medium is proposed, as shown in Figure 11. It has been widely accepted that the heterogeneous adsorption of biofilm plays a significant role in localized corrosion process of metal (Zhong et al., 2016; Jia et al., 2017; Gu et al., 2019). Generally speaking, biofilms can be considered as living cells trapped in a heterogeneous matrix containing extracellular polymeric substances (EPS), adsorbed organic and inorganic substances, interspersed with interstitial voids (Dou et al., 2018). Biofilms with stratified structure are never uniform and have different thickness and coverage at different positions of the metal surface, as shown in Figure 6A, 8A. There are different trends for bacterial density, bacterial activity and dissolved oxygen concentration with different biofilm thicknesses (Guo et al., 2017; Liu T. et al., 2018; Liu et al., 2019). Additionally, metabolic activity of bacteria in biofilm makes chemical environment near metal surface much different from that in bulk solution, such as ion species, concentration, pH, and oxygen level (Yuan and Pehkonen, 2007; Xu et al., 2013, 2016; Jia et al., 2018). These behaviors lead to electrochemical heterogeneity and induce or accelerate localized corrosion, as shown in Figure 6E, 8E,F. In *M*. *f*. medium, the metabolism of *M*. *f*. results in the decrease of pH and local acidification under biofilm, and there is a higher concentration of DO out of biofilm (Figure 1). This provides an advantage for initiation and progress of pitting corrosion, i.e., electrochemical active sites under biofilm and support of enough cathodic reaction out of biofilm. In mixed consortium medium (Figure 11), the metabolism of *M*. *f*. also results in local acidification in the inside of biofilm, which creates electrochemical active sites on surface of Q235 carbon steel. However, on the outside of biofilm, the metabolism of *T*. sp. leads to the decrease of DO concentration, which inhibits cathodic reaction, and results in a slower localized corrosion rate than that in *M*. *f*. medium (Figure 6E, 8F). The corrosion products, i.e., Fe^2+^ ions, are oxidized to Fe^3+^ ions during the metabolism of *M*. *f*., which react with H_2_O to form ferric oxides and H^+^ ions. Then, ferric oxides are used by *T*. sp. to form the Fe^2+^ ions, which react with FeOOH to produce magnetite (Fe_3_O_4_) (Ishikawa et al., 2002). Meanwhile, magnetite can be oxidized and reduced by *M*. *f*. and *T*. sp. (Weber et al., 2006). Therefore, the both RCs of Fe^3+^ and Fe^2+^ in corrosion products of Q235 carbon steel are between that in *M*. *f*. and *T*. sp. media (Figure 10 and Table 2).

## Conclusion

Corrosion of Q235 carbon steel is promoted in bacterial media due to the iron metabolism induced by *M*. *f*., *T*. sp., or mixed consortium. Corrosion rates of Q235 carbon steel in different culture media decrease in the order: *r*_*M.f.*_ > *r*_mixedconsortium_ > *r*_T.__sp._ > *r*_sterile_. The DO concentration decreases in both *T*. sp. and mixed consortium media, which results in the similar evolution of corrosion rate of Q235 carbon steel in these two media. Biofilm is locally adhered on Q235 carbon steel surface in both *M*. *f*. and mixed consortium media, which induces localized corrosion of Q235 carbon steel. Corrosion products components of Q235 carbon steel in mixed consortium medium are Fe_3_O_4_, FeOOH and Fe_2_O_3_, and their RCs are influenced by the presence of *M*. *f*. and *T*. sp.

## Data Availability

All datasets generated for this study are included in the manuscript and/or the supplementary files.

## Author Contributions

SC performed the experiments and the data analysis, and drafted the main manuscript. HD collated the experimental data. DZ and GL provided the place for experiments and modified the manuscript. All authors read and approved the final manuscript.

## Conflict of Interest Statement

The authors declare that the research was conducted in the absence of any commercial or financial relationships that could be construed as a potential conflict of interest.
